# Resolutions Adopted by the Odontological Society of Chicago in Reference to the Death of Dr. J. J. R. Patrick, of Belleville, Illinois

**Published:** 1895-08

**Authors:** 


### Resolutions adopted by the Odontological Society of Chicago, in reference to the death of Dr. J. J. R. Patrick, of Belleville, Illinois.

WHEREAS: Death has removed from our midst, Dr. John J. R. Patrick, of Belleville, Illinois, and there has passed from the scenes of human activity a character whose labors have enriched the stores of science, a man who delved fearlessly and steadfastly into those mysteries of nature which call forth the most subtle energies of the human mind, and yield results, making the lives of succeeding generations happier; now therefore be it

*Resolved*, That in the death of Dr. Patrick, the world loses an honored citizen soldier, the field of science a conscientious, faithful laborer, and the dental profession a light whose extinguishment will kindle the professional interest in the work he has accomplished for it, and thus extend and perpetuate his good influence. Be it further

*Resolved*, That to the family, friends and professional associates of Dr. Patrick, we extend the assurance that we appreciate his worth and are grateful that his life has been spared until it nearly completed the allotted time of three-score and ten.

JAMES A. SWASEY,
ALISON W. HARLAN,
LOUIS OTTOFY,
*Committee.*

